# Surface dose reduction from bone interface in kilovoltage X‐ray radiation therapy: a Monte Carlo study of photon spectra

**DOI:** 10.1120/jacmp.v13i5.3911

**Published:** 2012-09-06

**Authors:** James C. L. Chow, Amir M. Owrangi

**Affiliations:** ^1^ Department of Radiation Oncology Princess Margaret Hospital University Health Network Toronto ON Canada; ^2^ University of Toronto and Radiation Medicine Program Princess Margaret Hospital University Health Network Toronto ON Canada

**Keywords:** kilovoltage photon beam, kV X‐ray radiation therapy, Monte Carlo simulation, bone backscatter and surface dose

## Abstract

This study evaluated the dosimetric impact of surface dose reduction due to the loss of backscatter from the bone interface in kilovoltage (kV) X‐ray radiation therapy. Monte Carlo simulation was carried out using the EGSnrc code. An inhomogeneous phantom containing a thin water layer (0.5–5 mm) on top of a bone (thickness=1 cm) was irradiated by a clinical 105 kVp photon beam produced by a Gulmay D3225 X‐ray machine. Field sizes of 2, 5, and 10 cm diameter and source‐to‐surface distance of 20 cm were used. Surface doses for different phantom configurations were calculated using the DOSXYZnrc code. Photon energy spectra at the phantom surface and bone were determined according to the phase‐space files at the particle scoring planes which included the multiple crossers. For comparison, all Monte Carlo simulations were repeated in a phantom with the bone replaced by water. Surface dose reduction was found when a bone was underneath the water layer. When the water thickness was equal to 1 mm for the circular field of 5 cm diameter, a surface dose reduction of 6.3% was found. The dose reduction decreased to 4.7% and 3.4% when the water thickness increased to 3 and 5 mm, respectively. This shows that the impact of the surface dose uncertainty decreased while the water thickness over the bone increased. This result was supported by the decrease in relative intensity of the lower energy photons in the energy spectrum when the water layer was with and over the bone, compared to without the bone. We concluded that surface dose reduction of 7.8%–1.1% was found when the water thickness increased from 0.5–5 mm for circular fields with diameters ranging from 2–10 cm. This decrease of surface dose results in an overestimation of prescribed dose at the patient's surface, and might be a concern when using kV photon beam to treat skin tumors in sites such as forehead, chest wall, and kneecap.

PACS number: 87.55.K‐; 87.55.ne; 87.57.uq

## I. INTRODUCTION

In kilovoltage (kV) X‐ray radiation therapy, there is a dosimetric concern on certain skin treatment sites such as forehead, knee, and face where a thin layer of soft tissue is on top of a bone. When a skin tumor in the above sites is irradiated by kV photon beams, the loss of backscatter due to the bone inhomogeneity decreases the surface dose in the treatment. Since such dose reduction is not considered in the dose calculation based on the absolute dose calibration using a water phantom,^(1)^ an overestimation of dose occurs when it is prescribed. This leads to a dosimetric uncertainty with a variation of tissue thickness in the skin cancer treatment.

Measurements of surface dose reduction due to the loss of backscatter from inhomogeneities were carried out. Healy et al.^(2)^ measured the loss of backscatter from the lead for a Pantak Therapax superficial X‐ray machine (Elimpex‐Medizintechnik, Austria) using a thimble ionization chamber and solid water. They found that the dose perturbation at the phantom surface due to lead compared to air was minimal when the depth was larger than 3 cm. Butson et al.^(3)^ on the other hand, measured the dose reduction due to the bone inhomogeneity using a Gulmay D3300 X‐ray machine (Gulmay Medical Ltd., UK). Dose measurements were performed using an Attix parallel‐plate ionization chamber and EBT GAFCHROMIC film. Butson et al.^(3)^ found that the presence of bone affected backscattered dose by up to 12.5% of the surface dose, and GAFCHROMIC film could provide accurate dosimetric measurement for the surface dose reduction. Compared to the measurement, Monte Carlo simulation has the following advantages: (1) a very small voxel size (dose resolution) can be achieved with the voxel very close to the water–bone interface; (2) the geometric arrangements of the beam and phantom can be set up perfectly in the simulation program, which avoids any experimental human error in the measurement setup; and (3) there is no concern of the physical presence of the dosimeter body which would perturb the photon fluence in the experimental setup.

In order to investigate the impact of the dose reduction on the patient's surface due to the bone inhomogeneity, a detailed photon energy spectral study at the bone interface was carried out using an inhomogeneous bone phantom and Monte Carlo simulation. The photon energy spectra at the phantom surface and bone were determined with different water thicknesses. These energy spectra of photons and backscattered photons helped us to understand the relationship between the surface dose reduction due to the bone backscatter and water thickness in detail. In this study, a Gulmay D3225 X‐ray machine, which produces a photon beam with an energy of 105 kVp, was modeled for Monte Carlo simulations using the EGSnrc‐based BEAMnrc code.^4^, ^5^ The aim of this study is to investigate the dependence of the surface dose reduction on the water thickness due to the bone inhomogeneity when kV X‐ray radiation therapy is delivered to a skin tumor site of a thin layer of soft tissue in millimeter scale over a bone.

## II. MATERIALS AND METHODS

### A. Phantom and calculation geometry

**Figure 1:**
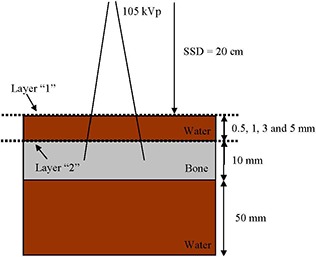
Schematic diagram (not to scale) showing the calculation configuration of the 105 kVp photon beam using the inhomogeneous bone phantom. The bone thickness is equal to 1 cm. The water and bone surface are represented by the horizontal broken lines of layers “1” and “2”, respectively.

An inhomogeneous phantom containing a thin water layer (0.5, 1, 3, and 5 mm) over a bone (thickness=1 cm) was used, as shown in Fig. 1. The phantom was irradiated by a kV photon beam perpendicular to the phantom surface. The density of bone is equal to $1.85\;g/{\text{cm}}^{3}$, and the bone (ICRPBONE521ICRU) contains elements of H, C, N, O, Mg, P, S, Ca and Zn in ratios of 4.69, 1.20, 0.29, 2.79, 0.0091, 0.34, 0.098, 0.52, and 0.00015, respectively.^(6)^ The bone was over a water layer of 50 mm to provide normal backscattering. The energy of the beam and source‐to‐surface distance (SSD) was equal to 105 kVp and 20 cm, respectively. The half‐value layer of the photon beam was 2 mm Al, and a filter of 2.4 mm Al was used. Treatment cones with diameters equal to 2, 5, and 10 cm were used to conform the beam field. Surface dose was calculated at the central beam axis on the phantom surface. The photon energy spectra were determined based on the particle scoring planes of layers “1” and “2” at the phantom surface and water–bone interface (Fig. 1), respectively. For comparison, all Monte Carlo simulations were repeated in a water phantom with the bone layer replaced by water.

### B. Monte Carlo simulation

The Electron Gamma Shower (EGSnrc) code (version 4‐r2‐2‐4) developed by the National Research Council Canada was used.^(6)^ In this code, the shape of the X‐ray energy spectra is improved by implementing the electron impact ionization model.^(7)^ Moreover, the efficiency of energy transition from the electron current to photons is increased by including the directional Bremsstrahlung splitting.^8^, ^9^

#### B.1 Phase‐space file of the kV photon beam

**Figure 2:**
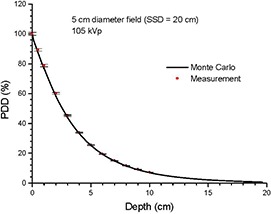
PDDs of the 105 kVp photon beam with circular field of diameter=5 cm and SSD=20 cm in water. The depth doses were calculated and measured using Monte Carlo simulation and parallel‐plate ionization chamber, respectively. The error bars show an uncertainty of 1%.

Photon beams of 105 kVp produced by a Gulmay D3225 X‐ray machine were used in this study. Open circular end fixed applicators with diameters of 2, 5, and 10 cm were used. The SSD was equal to 20 cm. The BEAMnrc^4^, ^5^ was used to generate phase‐space files based on the treatment head data of geometries and materials of different components such as the X‐ray tube, primary collimator, filter, ionization chamber, and applicator provided by the manufacturer. The treatment head model is the same as implemented by Knoos et al.^(10)^ The energy cutoff for electron and photon transport was set to 521 keV and 1 keV, respectively. Phase‐space files including information of the energy, orientation, type, charge, and position of particles crossing a scoring plane at the bottom of the applicator were generated containing about 36 million particles. The computing time was about 28 hours. Verification of the phase‐space beams was carried out by comparing the percentage depth dose (PDD) and beam profile in water calculated and measured by Monte Carlo simulation and parallel‐plate ionization chamber, respectively. Monte Carlo simulations were performed using a voxel phantom in DOSXYZnrc to determine the PDDs and beam profiles. Results of Monte Carlo simulation and measurement were found to agree well within a deviation of ±1% for circular fields of 2, 5, and 10 cm diameter. Figure 2 shows the PDD results from the Monte Carlo simulation and measurement for the circular field of 5 cm diameter.

#### B.2 Dose calculation using the DOSXYZnrc

Surface doses of the phantom in Fig. 1 were calculated using the DOSXYZnrc.^(11)^ The voxel size used in the simulation was $0.1\;\times \;0.1\;\times \;0.1\;{\text{mm}}^{3}$, which corresponding to the x‐, y‐, and z‐axis. One hundred million histories were run in each calculation per water thicknesses equal to 0.5, 1, 3, and 5 mm in the phantom of Fig. 1. In the simulation, PRESTA II was selected for the electron‐step algorithm.^6^, ^12^, ^13^ The spin effect, bound Compton scattering, Rayleigh scattering, atomic relaxation, and electron impact ionization were all set to “ON”. Since the low‐photon energies' electrons were not transported, the energy cutoff for electron and photon transport was set to 521 keV and 1 keV in the simulation to achieve a reasonable computing time, respectively.^10^, ^14^, ^15^ Under this approach, the relative dose error (statistical uncertainty as a fraction of dose in the voxel) was around 0.5% for all calculations.^11^, ^16^ In this study, surface doses of the inhomogeneous (Fig. 1) and homogeneous (bone replaced by water) phantoms were calculated.

#### B.3 Calculation of energy spectrum using the BEAMnrc and BEAMDP

The photon fluence at the phantom surface and bone was determined from the phase spaces which included the multiple crossers (backscattered photons) calculated by the BEAMnrc. The BEAMDP was used to calculate the energy spectrum based on the photon fluence.^(4)^ The number of bin in the photon energy spectrum was set to 200 in a range of 0–110 keV. Photon energy spectra at the phantom surface (layer “1”) and top of the bone (layer “2”) as shown in Fig. 1 were determined with water thicknesses equal to 0.5, 1, 3, and 5 mm. The SSD was kept constant (20 cm) when the water thickness was changed in the phantom. For comparison, Monte Carlo simulations were repeated in water phantoms with the same calculation configurations of the inhomogeneous phantoms.

## III. RESULTS & DISCUSSION

**Figure 3:**
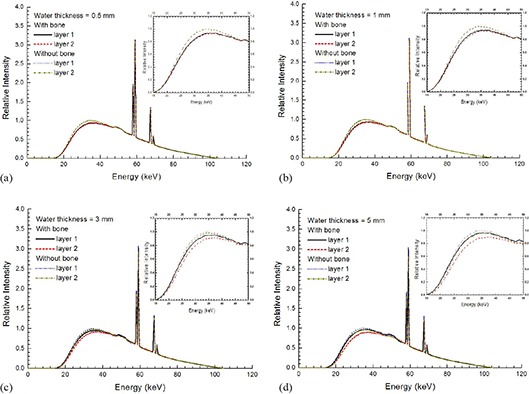
Relative photon energy spectra at layers “1” and “2” for the inhomogeneous bone (and water phantom using a circular field of 5 cm diameter. Water thicknesses are equal to (a) 0.5, (b) 1, (c) 3, and (d) 5 mm. The insets in the up the spectral curves in the energy range of 15–50 keV. All special curves were normalized to the maximum intensity of their Bremsstrahlung spectra.

**Figure 4:**
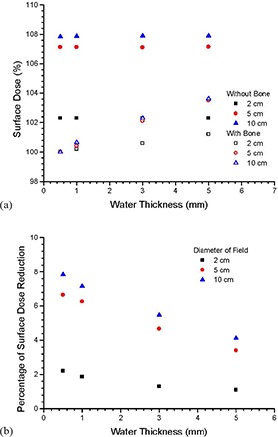
Dependence of the surface dose on the water thickness (a) in the inhomogeneous bone and water phantom, and percentage of surface dose reduction (b) varying with the water thickness for circular fields of 2, 5, and 10 cm diameter.

(Figures 3a), 3(b), 3(c), and 3(d) show the photon energy spectra at the water surface (layer “1”) and the bone (layer “2”) with water thicknesses equal to 0.5, 1, 3, and 5 mm using the circular field of 5 cm diameter, respectively. Photon energy spectra at the same surfaces using water phantom (without bone) are also included in Fig. 3. All spectral curves in Fig. 3 were normalized to the maximum intensity of their corresponding Bremsstrahlung spectra at layer “1” in the water phantom (i.e., without bone). The insets in Figs. 3 zoom up the spectral curves in the energy range of 15–50 keV, where intensities of the curves mostly varied there. (Figure 4a) shows the dependence of surface dose on the water thickness using the inhomogeneous bone and water phantom. Circular fields of 2, 5, and 10 cm diameter were used in (Fig. 4a), in which all doses are normalized to the surface dose of the inhomogeneous phantom with water thickness equal to 0.5 mm. (Figure 4b) shows the relationship between the percentage surface dose reduction and water thickness based on (Fig. 4a). The percentage surface dose reduction was determined by comparing surface doses with and without presence of the bone in the phantoms for different circular fields.

In (Figs. 3a)–(d), it is seen that the intensity of the photon spectra at the phantom surface is lower if the bone is present underneath the water. This decrease of photon fluence is evidence of the surface dose reduction due to the loss of backscatter from the bone. Moreover, it is found from these figures that the photon energy range responding the loss of backscatter is below about 50 keV (insets of Figs. 3). This shows that the loss of backscatter is mainly due to photons with lower energy in the spectrum. In (Fig. 3a), the intensities of spectral curves for layers “1” and “2” are very close and almost overlapped for the phantom with and without the bone. This is because the distance between the two layers is very small (0.5 mm) compared to (Figs. 3b)–(d). When the water thickness is increased to 1 mm, it can be seen in (Fig. 3b) that the difference between the intensity of spectral curves of layer “1” and “2” is still small. This difference, however, increases when the water thickness increases from 1 to 5 mm, showing that the effect of beam attenuation within the water layer becomes significant. In (Figs. 3b)and (3c), the intensity of the spectral curve for the layer “1” is found to be higher than that of the layer “2”. This shows that the depth dose is dominated by the beam attenuation. (Figure 4a) shows the surface dose varied with the water thickness in the inhomogeneous bone and water phantom. For circular fields of 2–10 cm diameter, it is seen in (Fig. 4a) that dose reduction occurs when bone is present underneath the phantom surface. Moreover, the surface dose increases with an increase of water thickness in the inhomogeneous bone phantom. The percentage of surface dose reduction due to the presence of the bone in (Fig. 4a) is shown in (Fig. 4b). For the circular field of 5 cm diameter, it is seen in (Fig. 4b) that when a bone is present underneath water, there is a surface dose reduction of 6.7% as the water thickness is equal to 0.5 mm. The dose reduction is decreased to 6.3%, 4.7%, and 3.4% when the water thickness is increased to 1, 3, and 5 mm. Similar trends of the surface dose reduction against water thickness can be found in the circular field of 10 cm diameter, and this shows that the impact of the surface dose uncertainty decreases when the water thickness above the bone increases. This agrees with the finding of Healy et al.^(2)^ In kV X‐ray radiation therapy, since the treatment dose at the patient's surface is prescribed by assuming the patient's body is homogeneous according to the absolute dose calibration, an underestimation of 6.7% to 3.4% of the prescribed dose might occur in the treatment sites such as the forehead, chest wall, and kneecap with soft tissue thickness equal to 0.5–5 mm using the circular field of 5 cm diameter. In addition, it is seen in (Fig. 4b) that the surface dose reduction becomes more significant when the field size is increased. This is because of the increased loss of scatter from a larger bone volume covered by a larger field. For a water thickness of 1 mm, the surface dose reduction varies between 1.8% and 7.2% for diameters of field ranged from 2 to 10 cm. This dosimetric uncertainty considerably and inevitably narrows down the total treatment uncertainty of 5%, including the uncertainties of patient setup, dose calibration, and dose calculation in the related skin radiation therapy.^17^, ^18^

Although there is no exact measurement published corresponding to our Monte Carlo simulations per the machine type, beam, and phantom geometry, Butson et al.^(3)^ have measured surface dose reductions for the circular field of 2 cm diameter with photon beam energy of 100 kVp. Surface dose reductions of 2%, 1.5%, and 1% were found with 0.5, 1, and 5 mm water thickness using their Gulmay D3300 X‐ray machine, respectively. These measurements can be compared to our Monte Carlo results of 2.2%, 1.8%, and 1.1% for the 0.5, 1, and 5 mm water thickness using a slightly higher 105 kVp photon beam, respectively.

## IV. CONCLUSIONS

In kV X‐ray radiation therapy on a thin layer of soft tissue over a bone, the dosimetric impact when the treatment dose is prescribed at the patient's surface was investigated using Monte Carlo simulation. The dose reduction due to the loss of backscatter from the bone was calculated by Monte Carlo simulation based on a clinical kV photon beam. In this study, photon beam energy of 105 kVp, field sizes of 2, 5, and 10 cm diameter and SSD=20 cm were used. For this circular field range, results showed that surface dose reductions of 7.8%–1.1% were found when distances between the phantom surface and bone were increased from 0.5–5 mm, due to the presence of the bone inhomogeneity. This surface dose reduction can be seen and explained from our photon energy spectral analysis, and can result in an overestimation of prescribed dose at the patient's surface. This dosimetric issue might be a concern when using kV photon beam to treat skin tumors in sites such as forehead, chest wall, and kneecap. Our future work includes studying variations of skin dose due to bone inhomogeneity using kV photon beams with different qualities.

## ACKNOWLEDGEMENTS

Photon beam dosimetry is based on the commissioning data of the Gulmay D3225 X‐ray machine carried out by Drs. M van Prooijen, A Beiki‐Ardakani, R Heaton and D Galbraith in the Princess Margaret Hospital. JCLC would like to thank Ms Amada Tulk from Gulmay Medical to allow us to share the Monte Carlo input data from Dr T Knoos in the Lund University Hospital for the verification of the photon beam. This study is supported by the Dean's Fund Grant in the University of Toronto.
